# Children with severe enterovirus A71 infection

**DOI:** 10.1186/s12887-023-03980-9

**Published:** 2023-04-13

**Authors:** Wenjuan Wu, Baoguang Li, Tao Xie

**Affiliations:** 1 Department of Neurology, Hebei Children’s Hospital, Hebei Children’s Hospital of Hebei Medical University, Hebei Provincial Key Laboratory of Pediatric Epilepsy and Neurological Diseases, 133 Jianhua Nan street, Shijiazhuang, 050031 China; 2 Department of Neurology, The Second Hospital of Hebei Medical University, Shijiazhuang, 050000 China

**Keywords:** Enterovirus, Nervous system, Cerebrospinal fluid, Imaging, Symptoms, Retrospective study

## Abstract

**Background:**

There are few reports on the timing of onset and the symptoms of enterovirus A71 (EV-A71) infection, which can easily be misdiagnosed. This study aimed to explore the clinical characteristics of children with severe EV-A71 infection.

**Methods:**

This retrospective observational study included children with severe EV-A71 infection admitted to Hebei Children’s Hospital between January 2016 and January 2018.

**Results:**

A total of 101 patients were included: 57 males (56.4%) and 44 females (43.6%). They were 1–13 years of age. The symptoms were fever in 94 patients (93.1%), rash in 46 (45.5%), irritability in 70 (69.3%), and lethargy in 56 (55.4%). There were 19 (59.3%) patients with abnormal neurological magnetic resonance imaging [pontine tegmentum (n = 14, 43.8%), medulla oblongata (n = 11, 34.4%), midbrain (n = 9, 28.1%), cerebellum and dentate nucleus (n = 8, 25.0%), basal ganglia (n = 4, 12.5%), cortex (n = 4, 12.5%), spinal cord (n = 3, 9.3%), and meninges (n = 1, 3.1%)]. There was a positive correlation between the ratio of neutrophil count and white blood cell count in cerebrospinal fluid in the first 3 days of the disease (r = 0.415, P < 0.001).

**Conclusion:**

The clinical symptoms of EV-A71 infection are fever and/or skin rash, irritability, and lethargy. Some patients have abnormal neurological magnetic resonance imaging. The white blood cell count in the cerebrospinal fluid of children with EV-A71 infection may increase alongside neutrophil counts.

## Introduction

EV-A71 is a non-enveloped icosahedron, single-stranded, positive-stranded RNA virus with a total genome length of 7.4 kb that was first isolated from the feces of children in California (USA) in 1969 and was detected in various countries since then [1–3]. Over the past 20 years, EV-A71 has also been widely detected in China [4, 5], causing outbreaks and several deaths [6–8]. The virus is neurotropic and can cause an inflammatory response in the body [9–11], leading to various neurological injuries and even death [12, 13].

There are many reports on the common clinical symptoms of infection by the EV-A71 virus in China [14–16], but there are few reports on the timing of onset and symptoms of children with severe EV-A71 infection, easily leading to misdiagnosis or misjudgment by clinicians, especially in rural hospitals, resulting in delayed treatment, improper treatment, or overtreatment.

Therefore, this study aimed to describe the clinical characteristics of children with severe EV-A71 infection.

## Materials and methods

### Study design and patients

This retrospective study included children with severe EV-A71 infection who were admitted to Hebei Children’s Hospital between January 2016 and January 2018. This study was approved by the Ethics Committee of Hebei Children’s Hospital (152). The requirement for informed consent was waived by the committee because of the retrospective nature of the study.

The inclusion criteria were (1) 28 days to 14 years of age, (2) a positive EV-A71-PCR throat swab, in line with the Guidelines for the Diagnosis and Treatment of Hand, Foot, and Mouth Disease (2018 version) [17], (3) one or more symptoms such as fever, rash, poor mental state, irritability, lethargy, limb weakness, ataxia, and cardiopulmonary failure, and (4) at least one symptom of alarm and lethargy. The exclusion criteria were (1) positive blood culture, (2) increased mycoplasma antibody titers, (3) elevated blood C-reactive protein (CRP) and procalcitonin, or (4) the physicians suspected the presence of other pathogens.

### Data collection

The general data of children with a severe EV-A71 infection were obtained from the electronic medical records of the hospital, including sex, age, fever, dermatosis, irritability, lethargy, limb weakness, white blood cell count, the proportion of neutrophils, white blood cell count of cerebrospinal fluid (CSF), and magnetic resonance imaging (MRI) of the head and/or spinal cord. The MRI examinations were performed using a GE Signa Excite 1.5-T imaging system during the study period. Two senior radiologists and pediatric neurologists with more than 10 years of experience analyzed the images.

### Statistical analysis

SPSS 23.0 (IBM, Armonk, NY, USA) was used to process and analyze the data. Continuous data with a normal distribution (according to the Shapiro-Wilk test) were expressed as means ± standard deviation and analyzed using Student’s t-test or the paired t-test. Categorical data were presented as n (%). Two-sided P-values < 0.05 were considered significant.

## Results

### Characteristics of the patients

This study included 101 patients: 57 males (56.4%) and 44 females (43.6%). They were 1 to 13 years of age (mean: 2.3 ± 1.7 years). Ninety-four (93.1%) patients had a fever, and the onset time was 1.1 ± 0.4 days. Forty-six patients (45.5%) had a rash, and the onset time was 1.1 ± 0.4 days. Seventy (69.3%) children had an irritable mood, and the onset time was 2.7 ± 2.3 days. Fifty-six (55.4%) children were lethargic, and the onset time was 3.8 ± 1.7 days. Two children (2.0%) displayed limb weakness, and the onset time was 5.0 ± 0.0 days. Two patients (1.98%) suffered from ataxia, and the onset time was 3.0 ± 0.0 days (Table 1). Two patients died on the 2nd and 4th days after onset, respectively.

**Table 1:** Clinical characteristics of the children with severe EV-A71 infection

	Cases	Time (days)
Sex	101	
Male	57	
Female	44	
Age, year	101	2.3 ± 1.7
Fever	94 (93.1%)	1.1 ± 0.4
Rash	46 (45.5%)	1.1 ± 0.4
Irritability	70 (69.3%)	2.7 ± 2.3
Lethargy	56 (55.4%)	3.8 ± 1.7
Limb weakness	2 (1.98%)	5.0 ± 0.0
Ataxia	2 (1.98%)	3.0 ± 0.0

### MRI characteristics

**Fig. 1:**
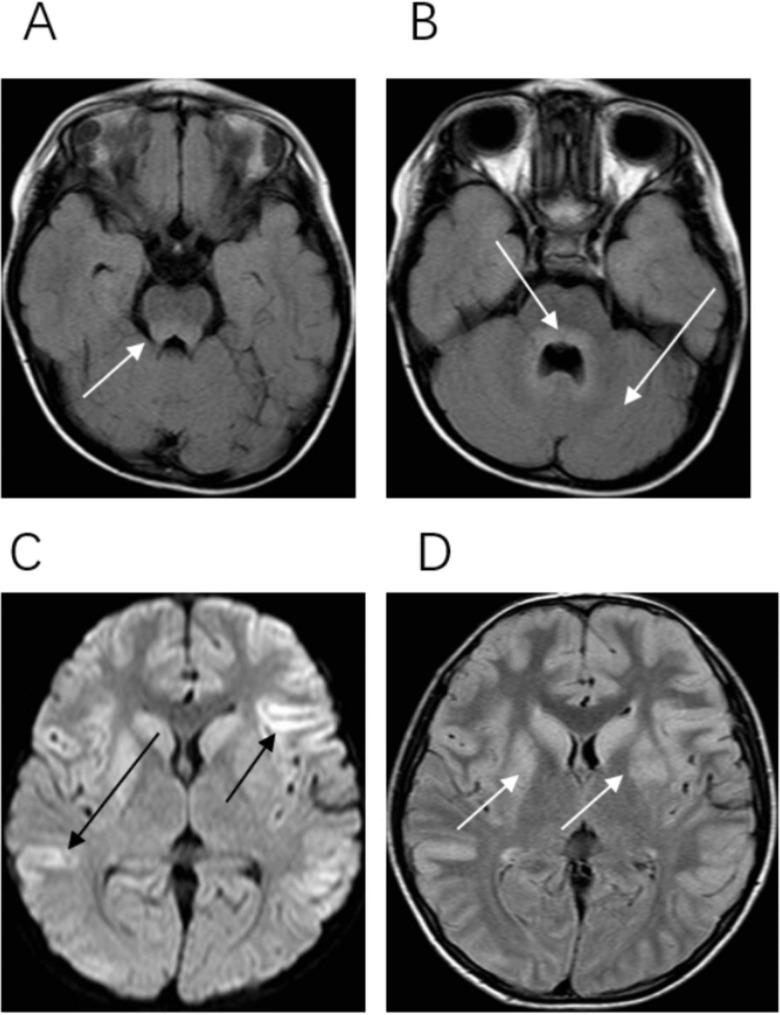
Typical brain imaging changes after infection of EV-A71. (A) Lesion in the midbrain. (B) Lesions in the dorsal pons and dentate nucleus. (C) Lesions in bilateral basal ganglia. (D) Multiple cortical lesions

Thirty-two children underwent an MRI of the head and/or spinal cord. The MRI examination was performed in the acute phase (within 1 week), and the average time of the MRI examination was 3.2 ± 2.8 days from the onset of the disease. Among the 32 children, 19 showed abnormal neurological imaging, accounting for 59.3%. The abnormal sites were pontine tegmentum (n = 14, 43.8%), medulla oblongata (n = 11, 34.4%), midbrain (n = 9, 28.1%), cerebellum and dentate nucleus (n = 8, 25.0%), basal ganglia (n = 4, 12.5%), cortex (n = 4, 12.5%), spinal cord (n = 3, 9.3%), and meninges (n = 1, 3.1%) (Fig. 1).

### Blood examinations

The white blood cell count within 3 days of the disease course was 5.7 × 10^9^/L to 25.9 × 10^9^/L. The proportion of neutrophils was 13.9–89.7%. Sixty-three patients underwent CSF examination within 3 days of the disease course. The white blood cell count in CSF was 0 to 587 × 10^6^/L (Table 2). The proportion of neutrophils within 3 days was divided into quintiles, with 20% per quintile. The proportions of neutrophils in the quintiles were positively correlated with the white blood cell count in CSF within 3 days (r = 0.415, P < 0.001) (Table 3).

**Table 2:** Examination of the children with severe EV-A71 infection

	Cases	‾x ± S
White blood cell count (×10^9^/L)	88	12.5 ± 4.2
Neutrophils (×10^9^/L)	88	53.2 ± 18.0
White blood cell count of cerebrospinal fluid (×10^9^/L)	63	114.8 ± 126.7

**Table 3:** Correlation between the proportion of neutrophils and white blood cell count of cerebrospinal fluid of 63 patients within 3 days of the disease course

Proportion of neutrophils within 3 days of the disease course	White blood cell count of cerebrospinal fluid within 3 days of the disease course (x ± S)
< 20% (n = 8)	45.1 ± 20.5
20-40% (n = 12)	83.5 ± 94.5
40-60% (n = 15)	115.3 ± 164.3
60-80% (n = 25)	125.7 ± 108.3
> 80% (n = 3)	331.7 ± 139.2

### Electrophysiology

**Fig. 2:**
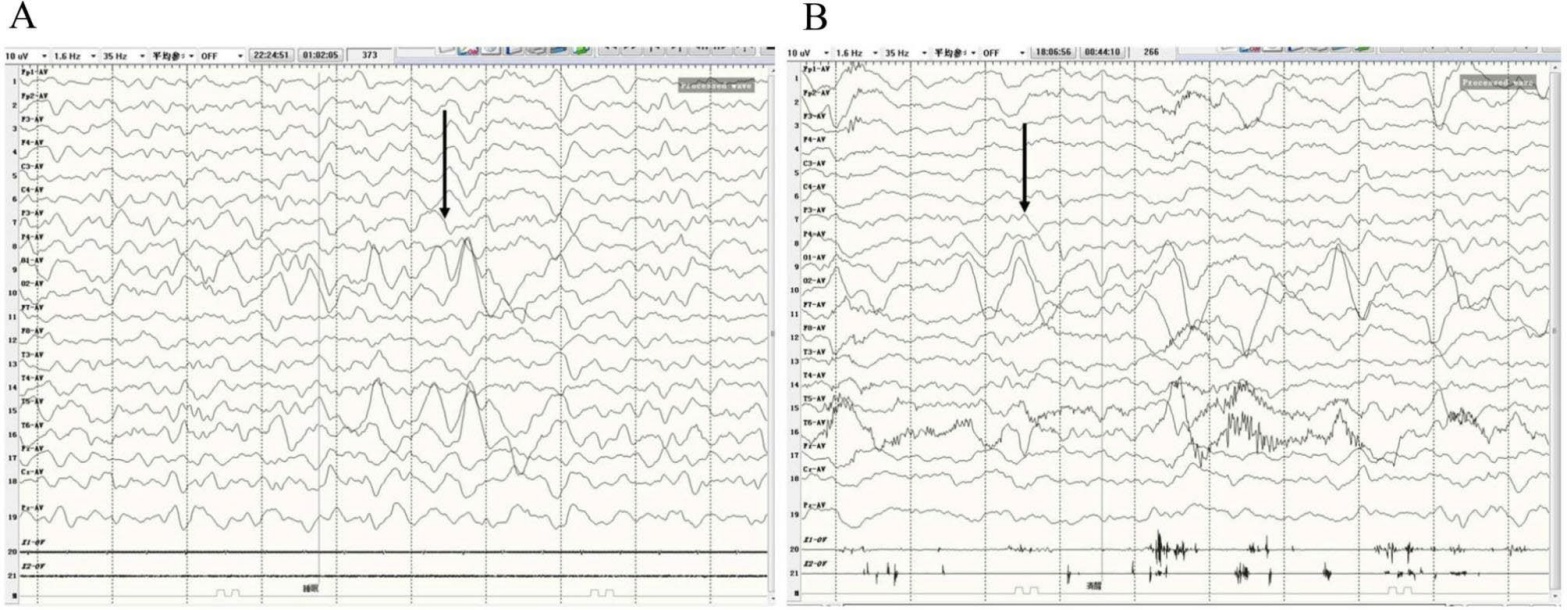
EEG manifestations caused by infection of EV-A71. (A-B) Background rhythm of EEG was slow, with 1.0-2.0-Hz slow waves

Forty patients were monitored using scalp electroencephalography (EEG) within the first 4 days of the disease. Among them, 37 (92.5%) children had a normal EEG. Three (7.5%) children showed slow waves of 1.0–2 Hz, and all three suffered from epilepsy-like seizures (Fig. 2).

## Discussion

This study showed that fever and rash tended to appear earlier during severe EV-A71 infection, followed by irritability and lethargy, which might be related to the infection mechanism. Indeed, EV-A71 enters the human body through the mouth, initially replicates in the pharynx (tonsils) or intestinal tract, and then multiplies in the regional lymph nodes, which can cause mild viremia [18–20]. The illness of most patients can be controlled at this stage. In some infected people, the virus continues to invade the reticuloendothelial tissue, deep lymph nodes, liver, spleen, bone marrow, skin, mucous membranes, central nervous system, and heart, and further proliferate and cause corresponding lesions [21]. Generally, the symptoms of systemic viremia, such as fever and rash, appear first after viral replication through the respiratory or gastrointestinal tract. If the disease continues to progress, the nervous system can be impaired, leading to irritability and lethargy.

In this study, irritability usually appeared on the 1st to 4th day, especially on the 2nd or 3rd day. The essence of irritability is myoclonus, divided into myoclonus from the corticothalamic axis and subcutaneous non-epileptic myoclonus originating from the back of the pons. Considering that no epilepsy waves were found in the scalp EEG of the included children and MRI lesions were more common in the dorsal pons, irritability may be from myoclonus caused by the release of 5-HT from the dorsal pons (predominant raphe nucleus) [22]. Lethargy mostly occurred after the fever subsided and after irritability, mainly on the 3rd or 4th day, and lasted for 1–2 days. Impairment of the cortical or ascending reticular activation system can reduce consciousness [23–25]. Still, most children had normal EEG without obvious cognitive dysfunction during the recovery period, suggesting likely non-specific projection system damage of the ascending reticular activation system in the upper pons or lower part of the midbrain, leading to lethargy.

Among the 101 children included, two died. None of the remaining children entered the stage of pre-cardiopulmonary or cardiopulmonary failure. Nevertheless, the frequencies of irritability and lethargy were 69.3% and 55.4%, respectively, suggesting that irritability and lethargy are common clinical manifestations of central nervous system involvement in most children with severe EV-A71 infection. In recent years, with the use of immunoglobulin, the number of cases of limb weakness has decreased compared with early studies. Limb weakness mostly occurred after the 4th day and resulted from the anterior horn of the spinal cord being invaded by the virus. The two cases of death due to cardiopulmonary failure occurred in the first 3 days of the course of the disease, mostly without skin rash. Considering that the lesions involved the dorsal nucleus of the vagus nerve and the nucleus of the solitary tract, or the inner acceleration center or vasoconstriction center of the reticular structure [26, 27], it is possible that the lesions were caused by sympathetic excitation.

MRI was performed on 32 patients in this study. The lesions involved pontine tegmentum (43.8%), medulla oblongata (34.3%), midbrain (28.1%), cerebellum and dentate nucleus (25.0%), basal ganglia (12.5%), cortex (12.5%), spinal cord (9.3%), and meninges (3.1%), consistent with the pattern reported by Lee et al. [28]. In this study, the incidence of radiographic infection sites was consistent with the incidence of the corresponding clinical manifestations in children: irritability (raphe nucleus), lethargy (ascending reticular activation system), ataxia (cerebellum and dental nucleus), limb weakness (anterior horn of the spinal cord), seizures (cortical) and meningeal irritation (meningeal).

Sixty-three patients underwent CSF examination within 3 days of the onset of the disease. The proportion of neutrophils was positively correlated with the white blood cell count in CSF within 3 days of the onset of the disease, suggesting that with the increase of the proportion, the white blood cell count in the cerebrospinal fluid has a gradually increasing trend, which has not been reported before and has certain significance for guiding the clinical cerebrospinal fluid examination of children with severe EV-A71 infection.

There were some limitations. Only one center was involved, leading to a small sample size. In addition, the numbers of patients with EV-A71 infection in the third and fourth stages were small. The retrospective nature of the study limited the data to those available in the charts. The mechanism of the EV-A71 virus invading the nervous system and the relationship between different mechanisms and the speed and severity of disease progression are not fully understood, and further research is needed.

## Conclusions

This study showed that the common clinical symptoms of EV-A71 infection were generally fever and/or rash, irritability, and lethargy. Some children were with abnormal neurological magnetic resonance imaging. There was a positive correlation between the ratio of neutrophil count and white blood cell count in CSF in the first 3 days of the course of the disease. EV-A71 infection may involve multiple parts of the nervous system, mainly the pontine tegmentum, consistent with corresponding clinical manifestations.

## Data Availability

All data generated or analyzed during this study are included in this published article.

## List of abbreviations

EV-A71: Enterovirus A71
CSF: Cerebrospinal fluid
